# The Extent of Extemporaneous Preparation and Regulatory Framework of Extemporaneous Compounding in Latvia

**DOI:** 10.3390/medicina55090531

**Published:** 2019-08-26

**Authors:** Olga Kiseļova, Baiba Mauriņa, Venta Šidlovska, Jānis Zvejnieks

**Affiliations:** 1 Department of Dosage Form Technology, Riga Stradins University, Dzirciema Street 16, LV-1007 Riga, Latvia; 2 State Agency of Medicines, Jersikas Street 15, LV-1003 Riga, Latvia

**Keywords:** extemporaneous preparations, community pharmacy, resolution, legislation, Latvia

## Abstract

*Background and objectives*: Extemporaneous preparations are pharmaceutical preparations individually prepared for a specific patient or patient group, but also high-risk products accompanied by doubts regarding their safety and quality. Legislation regulating the compounding of extemporaneous preparations is not harmonized among European countries. This problem is partially resolved by Resolution CM/Res(2016)1 on quality and safety assurance requirements for medicinal products prepared in pharmacies for the special needs of patients. In order to understand the relevance of extemporaneous compounding in Latvia and the fulfillment of the abovementioned resolution’s requirements, it is essential to get information about the volume and breakdown of sales of extemporaneous medicinal products in community pharmacies. The purpose of this survey is to identify the sales volume of extemporaneous preparations in community pharmacies in Latvia in 2017 by analyzing unpublished data of the State Agency of Medicines (SAM), as well as comparing Latvian laws with the requirements of the resolution. *Materials and Methods*: A separate Microsoft Excel spreadsheet was prepared for each statistical region in order to summarize the unpublished information of SAM on the turnover of extemporaneous preparations in 2017 in all Latvian statistical regions. In order to compare the regulatory framework in Latvia with the resolution, the Latvian Pharmaceutical Law and the Cabinet of Ministers Regulations regulating prescription, compounding and control of extemporaneous preparations in community pharmacies were analyzed. *Results*: Only 280 of 384 pharmacies submitted a report of sales of extemporaneous preparations for 2017 to the SAM. These pharmacies represented all Latvian statistical regions. Extemporaneous preparations were mostly sold in Riga (78.93%). The Latvian regulation does not include all paragraphs of the resolution. Most of the paragraphs of the resolution are described in Latvian regulatory enactments only partially. *Conclusions*: The total number of compounding pharmacies evidence that the service is needed. Latvian example highlights a necessity for European Union countries to compare their national legislation with the requirements of the resolution’s last version and, if necessary, implement relevant amendments.

## 1. Introduction

According to European Pharmacopoeia, extemporaneous preparations are pharmaceutical preparations individually prepared for a specific patient or patient group, supplied after preparation [1]. One of the main reasons for prescribing extemporaneous preparations is the non-existence of appropriate dosage forms of industrially manufactured medicines [2]. However, extemporaneous medicinal products are high-risk products accompanied by doubts regarding their safety, accuracy, shelf life and quality [3], because in comparison with industrially manufactured medicines, they are not subject to such strict quality control [4,5]. Several articles and reports were published reporting errors in the compounding of extemporaneous preparations in different countries [6,7,8,9]. 

The compounding of extemporaneous preparations developed differently all over the world, and there are no uniform standards with regard to compounding and control of these medicinal products even in European Union countries [10], and also legislation regulating the compounding of these medicinal products in pharmacies is not harmonized among European and US countries [11,12]. However, researchers emphasize that all European patients are entitled to receive medicinal products of equivalent quality and it is not permissible to apply different quality standards to extemporaneous preparations compounded in different European countries [11]. This problem is partially resolved by Resolution CM/Res(2016)1 on quality and safety assurance requirements for medicinal products prepared in pharmacies for the special needs of patients (hereinafter referred to as the resolution), which was first adopted by the Committee of Ministers of the Council of Europe in 2011 [12]. 

After the first adoption of the resolution a survey was carried out to determine how the resolution influenced national laws of several European countries [13]. This survey did not include data about Latvia. The resolution was updated in 2016. Since most of the Latvian regulatory enactments regulating the sector were adopted before the adoption of the resolution, a question arises, which paragraphs of the regulation are fulfilled in full or partially and which have not been fulfilled.

50.07% of community Latvian pharmacies had a special operation condition “preparation of medicinal products in the pharmacy” in the annex to their license in 2017. The fact that pharmacies are allowed to compound medicinal products does not mean that the relevant pharmacy exercises these rights. Information about the scope of sale of extemporaneous preparations in these pharmacies is not publicly available. In order to understand relevance of extemporaneous compounding in Latvia and fulfillment of the abovementioned resolution’s requirements, it is essential to get information about volume and breakdown of sales of extemporaneous medicinal products in community pharmacies.

The purpose of this survey is to identify sales volume of extemporaneous preparations in community pharmacies in Latvia in 2017 by analyzing unpublished data of the State Agency of Medicines (hereinafter referred to as the SAM), as well as comparing Latvian laws with the requirements of the resolution.

## 2. Materials and Methods

### 2.1. Sales Volume of Extemporaneous Preparations in Latvia

In accordance with order No. 271 of the Cabinet of Ministers “On Statistical Regions of the Republic of Latvia and Administrative Units Included in Them” Latvia is broken down into six statistical regions: Riga statistical region, Pieriga statistical region, Vidzeme statistical region, Kurzeme statistical region, Zemgale statistical region, Latgale statistical region. Furthermore, the regions consist of republican cities and municipalities [14]. 

A separate Microsoft Excel spreadsheet was prepared for each statistical region in order to summarize the unpublished information of SAM on the turnover of medicinal products compounded extemporaneously in 2017 in all Latvian statistical regions. Each Microsoft Excel spreadsheet summarizes these data about each community pharmacy that compounded medicinal products in the respective region:

- Name of the pharmacy;
- Name of the company owning the pharmacy;
- Municipality or republican city, where the pharmacy is located;
- Amount of money (in euro, without VAT), which the pharmacy obtained from the sale of extemporaneous medicinal products to natural and legal persons;
- Share in percentage of the total amount of money (in euro, without VAT), which the pharmacy obtained from the sale of extemporaneous preparations to natural and legal persons. As the total sales volume of all Latvian community pharmacies for extemporaneous preparations (in euro, without VAT) is known, each pharmacy’s share is expressed as a percentage from total sales volume. Hospital pharmacies were not included in the study.

### 2.2. Compliance of Latvian Laws with the Requirements of the Resolution

In order to compare regulatory framework in Latvia with the resolution, the Latvian Pharmaceutical Law and the following Cabinet of Ministers Regulations regulating prescription, compounding and control of extemporaneous preparations in community pharmacies were analyzed:

- Regulations of the Cabinet of Ministers No. 377 of 31 May 2005 “Procedure of Circulation of Alcohol in Pharmaceutical Companies, Veterinarian Pharmaceutical Companies, Pharmacies, Medical Institutions and Veterinary Medicine” as amended;
- Regulations of the Cabinet of Ministers No. 57 of 17 January 2006 “Regulations Regarding Procedures for the Labelling of Medicinal Products and the Requirements to Be Set for the Package Leaflet of Medicinal Products” as amended;
- Regulations of the Cabinet of Ministers No. 304 of 18 April 2006 “Regulations Regarding the Procedures for the Manufacture and Control of Medicinal Products, the Requirements for the Qualification and Professional Experience of a Qualified Person and the Procedures for the Issuance of the Certificate of Good Manufacturing Practice to a Medicinal Products Manufacturing Undertaking” as amended;
- Regulations of the Cabinet of Ministers No. 376 of 9 May 2006 “Procedures for the Registration of Medicinal Products” as amended;
- Regulations of the Cabinet of Ministers No. 416 of 26 June 2007 “Procedures Regarding the Distribution and Quality Control of Medicinal Products” as amended;
- Regulations of the Cabinet of Ministers No. 288 of 23 March 2010 “Regulations Regarding Operating of Pharmacies” as amended;
- Regulations of the Cabinet of Ministers No. 610 of 2 August 2011 “Criteria for the Location of Pharmacies and Pharmacy Branches” as amended;
- Regulations of the Cabinet of Ministers No. 800 of 19 October 2011 “Procedures for the Licensing of Pharmaceutical Activity” as amended;
- Regulations of the Cabinet of Ministers No. 344 of 25 June 2013 “Procedures for Importing and Distributing Active Substances” as amended.

### 2.3. Ethical Approval

The study “Availability of extemporaneous preparations in Latvian pharmacies: a quantitative and qualitative assessment of the situation and prospect for the future” was allowed by the Ethics Committee of Riga Stradins University (Identification code Nr. 14, date of approval 05.10.2017.)

## 3. Results

### 3.1. Overview of Latvian Pharmacies, Which Had a Special Operation Condition “Preparation of Medicinal Products in the Pharmacy” in the Annex to Their Licence in 2017

An accurate number of extemporaneous medicinal products prepared in Latvian pharmacies based on prescriptions issued by medical practitioners in 2017 is unknown, however, data about the market share of extemporaneous preparations are available. In accordance with SAM data, extemporaneous preparations account for a small market share compared to finished medicinal products, including prescription medicines and non-prescription medicines (only 0.65%). Zero point six-five percent, or 2.25 million euros (without VAT) was the total sales volume of all Latvian community pharmacies for extemporaneous preparations in 2017, but the breakdown of 2.25 million euros by Latvian pharmacies is uneven. Thus each pharmacy’s share is expressed as a percentage from 2.25 million euros. In 2017, 384 pharmacies or 50.07% of all the community pharmacies had a special operation condition "preparation of medicinal products in the pharmacy" in the annex to their license. Although many pharmacies were entitled to prepare medicinal products, the data below show that this service was not provided by all the pharmacies. Every year the pharmacies compounding medicinal products should submit to the SAM a report stating the amount of money (in euro, without VAT), which the pharmacy obtained from the sale of extemporaneous medicinal products to natural and legal persons.

**Figure 1:**
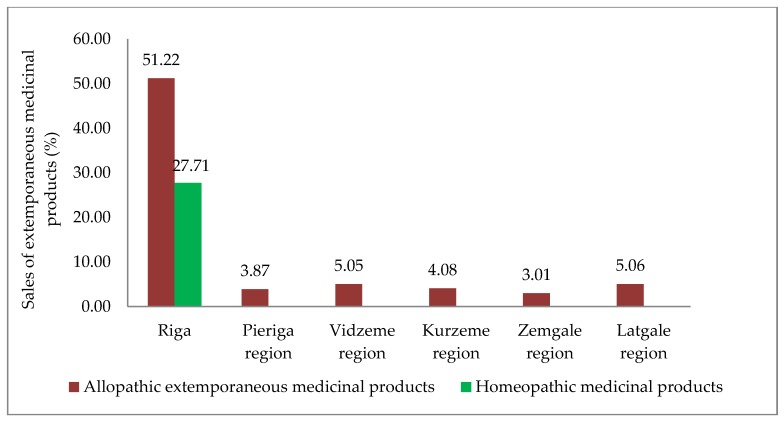
Sales of extemporaneous medicinal products in Latvian statistical regions in 2017 (%).

Only 280 of 384 pharmacies submitted a report of sales of extemporaneous preparations for 2017 to the SAM. These pharmacies represented all Latvian statistical regions: Riga statistical region (108 pharmacies), Pieriga statistical region (39 pharmacies), Vidzeme statistical region (32 pharmacies), Kurzeme statistical region (35 pharmacies), Zemgale statistical region (27 pharmacies), Latgale statistical region (39 pharmacies). Extemporaneous medicinal products were mostly sold in Riga, and not only allopathic extemporaneous medicinal products, but also homoeopathic medicinal products were compounded in Riga (Figure 1).

Pharmacies in Riga represented 12 different limited liability companies (hereinafter LLC) and 1 joint stock company (hereinafter JSC). The breakdown of extemporaneous preparations in these pharmacies is uneven. In 27 pharmacies, the share in the percentage of sales of extemporaneous medicinal products in total sales was lower than 0.01%. In 73 pharmacies it was within 0.01% to 0.44%. Only eight pharmacies crossed the 1% barrier and their sales were within 1.22% to 23.11%. 

In the Pieriga statistical region the pharmacies were located in 1 republican city (Jurmala) and in 16 municipalities. The pharmacies in Pieriga represented 8 different LLCs and 1 JSC. None of the pharmacies crossed the 1% barrier. In seven pharmacies, the share in percentage of sales of extemporaneous medicinal products in total sales was lower than 0.01%, in other 32 pharmacies it was within 0.01% to 0.72%. 

In the Vidzeme statistical region the pharmacies were also located in one republican city (Valmiera) and in 16 municipalities. The pharmacies represented 14 different LLCs and 1 JSC. In four pharmacies, the share in percentage of extemporaneous medicinal products in total sales was lower than 0.01%. One pharmacy crossed the 1% barrier, its sales were 1.78%. In other pharmacies it was within 0.01% to 0.78%. 

In the Kurzeme statistical region the pharmacies were located in two republican cities (Liepaja and Ventspils) and in six municipalities. The pharmacies represented seven different LLCs and one JSC. In nine pharmacies, the share in percentage of extemporaneous medicinal products in total sales was lower than 0.01%. Similarly to the Vidzeme statistical region, only one pharmacy crossed the 1% barrier, its sales were 1.57%. In other pharmacies it was within 0.01% to 0.82%. 

In the Zemgale statistical region the pharmacies were located in two republican cities (Jelgava and Jekabpils) and in nine municipalities. The pharmacies represented 11 different LLCs and 1 JSC. None of the pharmacies crossed the 0.5% barrier. In four pharmacies, the share in percentage of extemporaneous medicinal products in total sales was lower than 0.01%. In other pharmacies it was within 0.01% to 0.48%. 

In the Latgale statistical region the pharmacies were located in two republican cities (Daugavpils and Rezekne) and in nine municipalities. The pharmacies represented 10 different LLCs, one JSC and one individual merchant (hereinafter IM). In nine pharmacies, the share in percentage of extemporaneous medicinal products in total sales was lower than 0.01%. Two pharmacies in Daugavpils crossed the 1% barrier, their sales were 1.09% and 1.08%. In other pharmacies it was within 0.01% to 0.65%. 

**Figure 2:**
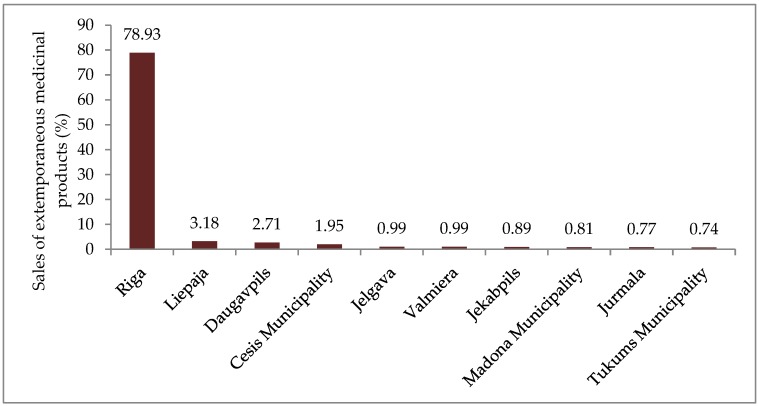
10 republican cities and municipalities, where sales of extemporaneous medicinal products in 2017 were the highest (%).

The data show that apart from Riga main sales of extemporaneous medicinal products were observed in two republican cities (Liepaja, Daugavpils) and 1 municipality (Cesis Municipality), however in much smaller amounts than in Riga. In other republican cities and municipalities income from sales of medicinal products made in pharmacies were lower than 1% of total sales of extemporaneous medicinal products (Figure 2).

### 3.2. Compliance of Latvian Laws with the Requirements of the Resolution

The resolution consists of 13 paragraphs, first of which explains the field of application of the resolution, the second explains the definitions used in the resolution, while Paragraphs 3–13 describe the requirements for the quality and safety assurance of medicinal products prepared in pharmacies [12].

In accordance with Paragraph 3 “added value of pharmacy preparations and responsibilities of health care professionals” of the resolution a pharmacist should check whether the prescribed pharmacy preparation has a suitable industrially manufactured equivalent available on the national market. This is partially described in CM Regulations No. 288, which provide that: “if the prescribed medicinal product is not available in a ready-made form of medicinal product, the pharmacist shall ensure the preparation thereof” [15]. Latvian laws do not stipulate replacement of extemporaneous preparations with industrial preparations, and neither the Pharmaceutical Law nor the CM regulations set a limit that a pharmacist is allowed to prepare only the medicinal products, which have no industrial equivalent. A patient may submit a prescription for an extemporaneous preparation to any community pharmacy in Latvia. All community pharmacies, whose licences do not include a special operation condition “preparation of medicinal products in the pharmacy” in their annex, should conclude an agreement on the preparation of the medicinal product with a pharmacy, which is offering this service. The prepared medicinal product is delivered to the pharmacy, to which the patient has submitted his or her prescription [15]. CM Regulations No. 304 provides that: “the head of a pharmacy shall be liable for the quality of medicinal products prepared in the pharmacy” [16]. This sentence provides that the medicinal product preparing pharmacy is responsible for the quality of appropriate product.

As to Paragraph 4 “preparation process” of the resolution, Latvian regulatory enactments do not currently contain requirements for the Good Manufacturing Practices Guide (hereinafter referred to as the GMP Guide) and the Guide to Good Practice for the Preparation of Medicinal Products in Health Care Establishments in Pharmaceutical Inspection Convention and Pharmaceutical Inspection Co-operation Scheme (PIC/S GPP Guide) for medicinal products prepared in community pharmacies, but the implementation of the PIC/S GPP Guide is planned in CM Regulations No. 288. The Latvian Language Agency translated the PIC/S GPP Guide into Latvian in 2017 [17]. The quality of medicinal products compounded in a pharmacy is supervised by SAM, and its operational strategy for 2017–2019, section on the improvement of competences of SAM employees, emphasises the need to increase competence in relation to the PIC/S standard [18]. Although the implementation of the above-mentioned documents in Latvia is just a plan, currently valid regulatory enactments [15,16] contain paragraphs partially correlating with requirements of Paragraph 4 of the resolution—when accepting a prescription and also when compounding extemporaneous preparations, the composition of the extemporaneous preparations should be evaluated to ensure safety and efficacy of the medicinal product. A pharmacist should observe compatibility and physical and chemical properties of substances, as well as principles of pharmaceutical technology. The requirements to the arrangement and location of premises for compounding of extemporaneous preparations are determined by CM Regulations No. 288. These premises depending on the specifics of pharmacy prescriptions should have arranged and equipped workplaces to prepare and analyse liquid, semi-solid and solid dosage forms. Aseptic conditions should be provided for the preparation of sterile dosage forms [15]. Education requirements to pharmacy staff involved in compounding and control of extemporaneous preparations are laid down by the Pharmaceutical Law, where the rights to perform these actions are granted only to specialists having pharmaceutical education—pharmacists and pharmacists’s assistants [19]. CM Regulations No.288 provide that the duty of the head of a pharmacy is to provide the pharmacy with appropriately qualified employees [15].

At present, Latvian regulations do not contain the requirement regarding the creation of a product dossier for extemporaneous medicinal products as referred to in Paragraph 5. They also do not include the risk assessment of extemporaneous medicinal products recommended in Paragraph 5.2 “Risk assessment of a pharmacy preparation” consisting of two levels (“high-risk preparations” and “low-risk preparations”). In accordance with CM Reg. No. 304 a pharmacist, when accepting a prescription for compounding, shall study the prescribed composition, which includes compatibility of components, check of doses of substances having strong effect, as well as shall ascertain that maximum amounts of ethyl alcohol, narcotic and equivalent psychotropic substances that can be prescribed with one prescription have not been exceeded [16,20]. In accordance with the Pharmaceutical Law, the SAM shall evaluate and check compliance of manufacturers and importers of the active substance with GMP, and shall issue GMP certificates [19]. Furthermore, CM Regulations No. 344 provide that only those active substances can be used in the preparation of medicinal products, which were purchased from manufacturers and distributors registered with SAM [21].

Paragraph 5.3 of the resolution describes the availability of data for authorities for inspection or upon request. CM Regulations No. 304 provides that the Health Inspectorate shall conduct inspections in the pharmacies preparing medicinal products at least once a year. The Health Inspectorate is entitled to send samples of the extemporaneous preparations compounded, the purified water obtained, the concentrates and semi-finished products to be used for the compounding of extemporaneous preparations in a pharmacy to a laboratory for examination, including for microbiological testing, if there are doubts about their quality. Pharmacies shall document the process of preparation and analysis of medicinal product by making entries in the log for registration of testing results for medicinal products prepared based on individual prescriptions or upon request of medical institutions; the log for registration of testing results for purified water obtained in a pharmacy, the log for registration of testing results for concentrates, semi-finished products, ethyl alcohol; the log for registration of testing results of identity of raw materials [16].

The marketing authorisation referred to in Paragraph 6 of the resolution has not been introduced in Latvia. Pursuant to the Pharmaceutical Law and CM Regulations No. 376, the medicinal products compounded for an individual patient do not require registration at SAM [19,22].

Labelling of extemporaneous medicinal products generally meets the requirements of Paragraph 7 “Labelling” of the resolution. CM Regulations No. 57 provide a detailed description of that. Unlike in the labelling of finished dosage forms, warnings are listed, which are added to labelling when needed, for example, “Shake before use”. Latvian regulations do not include the requirements that labelling should contain information not only about the pharmacy, in which the medicinal product was prepared, but also should state the name, address and telephone number of the pharmacy, where the medicinal product was ordered and issued [23].

Paragraph 8 of the resolution is devoted to “Compliance with pharmacopoeial requirements”. Latvia has no up-to-date version of national pharmacopoeia, as well as there are no officially approved instructions and quality standards for preparation of medicinal products in a pharmacy. CM Regulations No. 344 provides that only those active substances can be used in the compounding of extemporaneous preparations, which were purchased from manufacturers and distributors registered with SAM. Active substances should be produced in accordance with principles of good manufacturing practice and guidelines [21]. CM Regulations No. 288 provide that it is the duty of the head of a pharmacy to draft instructions for compounding and control of extemporaneus preparations, while the pharmacist’s task is to compound extemporaneous preparations in accordance with the instructions approved by the head [15]. Having carried out quality control of the compounded medicinal product, a pharmacist issuing the medicinal product shall check compliance of packaging of the medicinal product with physical and chemical properties of the components included in the medicinal product [16].

Latvian regulatory enactments do not provide for reconstitution of medicinal products in health care establishments in accordance with Paragraph 9 of the resolution.

The Latvian regulations meet the requirements of Paragraph 10 “Authorisation for pharmacies or licences for companies making preparations for pharmacies” of the resolution. In accordance with CM Reg. No. 800 a licence should be received to open a community pharmacy. A licence for opening (operation) of a pharmacy is issued by SAM, and it is entitled to suspend and renew the licence [24]. In Latvia preparation of extemporaneous medicinal products is within the competence of pharmacies only. The Pharmaceutical Law defines preparation of medicinal products as a component of pharmaceutical care [19]. In order to compound extemporaneous preparations, a pharmacy should receive permission from SAM, which specifies the special operation condition “preparation of medicinal products in the pharmacy” in the annex to the licence [24]. Community pharmacies may prepare medicinal products for an individual patient based on individual prescriptions or upon a written request of a medical institution [19]. Since the compounding of extemporaneous preparations is an additional service of a pharmacy and not all Latvian pharmacies offer this service, the Latvian laws support the pharmacies preparing medicinal products. To protect these pharmacies, CM Regulations No. 610 provide that when a pharmacy is moved, it cannot be located within a 500-metre radius of other community pharmacy, which prepares medicinal products [25]. This restriction for movement of pharmacies was first set in 2002 [26]. Since then the number of pharmacies having a special operation condition “preparation of medicinal products in the pharmacy” in the annex to their licence has increased more than three times—120 pharmacies in 2003, 422 pharmacies in 2019 [27,28]. The pharmacies, which do not compound extemporaneous preparations, should conclude an agreement on the compounding of the medicinal product with a pharmacy, which has this special operation condition in the annex to its licence [15]. 

Paragraph 11 of the resolution is devoted to “Transparency and safety”. As it was mentioned before, in accordance with CM Reg. No. 304, the process of preparation and analysis of medicinal products shall be documented by pharmacies. The Health Inspectorate shall inspect the pharmacies preparing medicinal products at least once a year [16]. SAM has information about all the pharmacies preparing medicinal products, lists of these pharmacies are published on a regular basis [28]. At present, SAM does not have accurate data about the full composition of the available pharmacy preparations and preparing pharmacies’ portfolio of different preparations. However, the State Agency of Medicines has taken measures to obtain this information asking pharmacies to send compositions of prescriptions, which are compounded most often. Compositions of medicinal products prescribed by physicians and compounded by pharmacies are not subject to clinical expertise. In accordance with the Pharmaceutical Law, the Health Inspectorate is entitled to prohibit the distribution of any medicinal products, active substances and excipients, if they have been found to be of inferior quality or falsified, but in a case, when doubts have arisen regarding their quality—to suspend distribution of the relevant medicinal products, active substances or excipients pending a final clarification of their quality [19].

The requirements of Paragraph 12 “Communication and information to patients” of the resolution are included in CM Regulations No. 304 and 57. The persons issuing medicinal products must check labelling of compounded extemporaneous preparations [16]. When issuing extemporaneous preparations, a pharmacist shall explain how to use and store the compounded extemporaneous preparation, as well as emphasises that this medicinal product can be used only during the period indicated by the physician and until their shelf life is over. Medicinal product administration conditions (dosage, route and frequency of administration), and special storage conditions, as well as shelf life are always specified on the labelling of compounded medicinal products [23], therefore the patient receives this information orally and in writing.

Paragraph 13 “Distribution of pharmacy preparations” of the resolution is partially mentioned in the Pharmaceutical Law and CM Regulations No. 416. SAM’s duties include the evaluation of compliance of distributors of medicinal products and active substances with good distribution practices and issuing of good distribution practice certificates [19]. Meanwhile, CM Regulations No. 416 provide that: “in order to monitor the implementation of and compliance with the good distribution principle, the head of the pharmacy shall conduct self-control and shall record measures of the self-control” [29]. The Latvian law does not describe requirements for export and import of preparations.

The full implementation of the resolution is time consuming as it requires revision of the above mentioned Latvian laws and amendments of the Cabinet of Ministers. However, there are some activities that are relatively easy to implement, but effective. One of them is the implementation of PIC/S GPP Guide in national legislation of Latvia, which is currently in process. The number of non-standardized extemporaneous formulations could be reduced by the norm to extemporaneously prepare only those products who have not a suitable industrially manufactured equivalent available on the national market and creation of a database of compositions of medicinal products prescribed and compounded most often.

## 4. Discussion

The share of community pharmacies compounding extemporaneous preparations varies across European countries. For example, unlike in Latvia, all community pharmacies in Portugal and Germany offer the service “preparation of medicinal products in a pharmacy” [30,31]. While the preparation of extemporaneous medicinal products in Denmark is centraliszed in three community pharmacies [32]. 

Sales of extemporaneously prepared medicinal products in Latvia in community pharmacies are low (0.65%) compared to industrially manufactured medicinal products. Similar data were obtained also in other European countries. For example, in Finland, where dispensed extemporaneous preparations account for 0.5% of all medicines [33]. Also in Spain, non-sterile extemporaneous preparation account for only about 2% of all the medicines dispensed based on prescriptions [34]. 

Main sales of extemporaneous medicinal products were observed in Riga, the capital of Latvia. This could be explained by data from Central Statistical Bureau of Latvia—at the beginning of 2017 the largest number of Latvian residents lived in Riga (32.9%) [35]. Also, the largest number of physicians was employed in Riga—62% of the total number of all Latvian physicians, in other regions significantly less (6–9%) [36]. 

The Latvian regulation does not include all paragraphs of the resolution. Most of the paragraphs of the resolution are described in Latvian regulatory enactments only partially. This may be explained by the fact that in the majority of cases the CM Regulations regulating preparation of medicinal products in a pharmacy were adopted before the adoption of the resolution. Time is required to introduce changes to currently applicable regulations. Similar data were also obtained in the survey regarding the impact of the resolution adopted in 2011 on the legislation of 12 European countries. Although most of the countries did not fulfill all paragraphs of the resolution, the author has concluded that: “the overall results of the survey indicate that among the countries involved there is, in general, a clear commitment to implement the recommendations of the Resolution”. The paragraphs, which are fully described in Latvian regulatory enactments, are mainly described also in other European countries. For example, Belgium, the Czech Republic, Denmark, Finland, Ireland, Italy, the Netherlands, Poland, Portugal, Serbia, Switzerland and the United Kingdom like us comply with recommendations mentioned in paragraph “Labelling”. The paragraph of the resolution named “Marketing authorization” is not included in the Latvian regulatory enactments. Other European countries face a similar situation. The above-mentioned survey revealed that only one of 12 countries included in the survey partially implemented recommendations about marketing authorization [13].

## 5. Conclusions

- The survey results demonstrate that the service “preparation of medicinal products in a pharmacy” is offered in all Latvian statistical regions. The total number of compounding pharmacies (280 or 36.5% of all community pharmacies) evidence that the service is needed. The income of pharmacies of different regions from sale of extemporaneous preparations compounded by them shows that most of the extemporaneous medicinal products are compounded in the Riga statistical region (78.93%) and amounts in other regions are considerably smaller.
- Latvia implemented recommendations of the Council of Europe only partially. Latvian regulatory enactments meet Paragraphs 7, 10, 12 of the resolution. Paragraphs 3, 4, 5, 8, 11, 13 of the resolution are partially described in the Latvian regulatory enactments. Paragraphs 6, 9 of the resolution are not described in the Latvian regulatory enactments. The Latvian example highlights a necessity for European Union countries to compare their national legislation with the requirements of the resolution’s last version and, if necessary, implement relevant amendments.
